# Urinary incontinence and sexual health in a population sample of older people

**DOI:** 10.1111/bju.14177

**Published:** 2018-04-05

**Authors:** David M. Lee, Josie Tetley, Neil Pendleton

**Affiliations:** ^1^ Faculty of Health, Psychology and Social Care Manchester Metropolitan University Manchester UK; ^2^ Institute of Brain, Behaviour and Mental Health University of Manchester Salford UK

**Keywords:** urinary incontinence, sexual health, sexual function, population‐based, retrospective, ELSA, #Incontinence

## Abstract

**Objectives:**

To investigate the association between self‐reported urinary incontinence (UI) and sexual health in a representative sample of older people.

**Subjects and Methods:**

Participants were community‐dwelling women and men aged 50–90+ years from the English Longitudinal Study of Ageing (ELSA) who reported any sexual activity in the last year. The prevalence of UI was assessed both cross‐sectionally (ELSA Wave 6; 2012) and retrospectively over the preceding 8 years (ELSA Waves 2–6; 2004–2012). Sexual activities, difficulties and concerns were assessed using a validated Sexual Relationships and Activities Questionnaire. The association between UI and sexual health outcomes was examined using weighted logistic regressions, with adjustments made for demographic, health, and lifestyle factors.

**Results:**

At Wave 6, 391 (20.0%) women and 141 (6.9%) men reported ‘any UI’ in the last 12 months. Compared to those without UI, women with UI reported declines in sexual activity and arousal over the last year, and increased concern about their frequency of sexual activity and ability to become sexually aroused. Men with ‘any UI’ reported declines in sexual desire, increased erectile and orgasm difficulties, and were more concerned about these sexual functions compared to men without UI. Differences in the patterns of association with sexual health were seen, dependent upon whether UI was reported as sporadic or persistent, and also with respect to the duration of retrospectively reported UI.

**Conclusion:**

Self‐reported UI was associated with impairment in sexual health in women and men, and mainly linked to recent declines in sexual activity and function along with elevated sexual concerns. Our findings highlight that the sexual health of older people should be considered when managing UI.

## Introduction

Urinary incontinence (UI), defined as the complaint of any involuntary leakage of urine 1, is a common condition that becomes more prevalent with increasing age 2. Typically, UI is categorised as stress UI, urgency UI, or mixed UI 3. In addition, overactive bladder (OAB) is associated with urinary urgency, with or without urgency UI symptoms 4. The prevalence, incidence and natural history of UI have been extensively reported in the literature 5, 6, 7, and correlates and risk factors have been identified in numerous epidemiological studies 8, 9, 10, 11. Although estimates are variable and not consistently comparable between studies, the prevalence of UI in women gradually increases through adulthood, with 9–39% of women aged >60 years reporting ‘daily UI’ 2. Data for men consistently show a lower prevalence, with estimates ranging from 2% to 11% for daily UI amongst men aged ≥65 years 2.

Whilst not life threatening, UI is associated with significant morbidity and can have a marked negative impact on health‐related quality of life (QoL) and affect both psychological and social well‐being 4, 9, 12, 13. Sexual health and satisfaction are increasingly recognised as positive indicators of QoL and emotional well‐being in both men and women 14, 15, 16, 17. Although previous studies have described the negative impact UI has on older peoples’ sexual lives and satisfaction, they have tended to be: focused on patients attending genitourinary clinics 18; based on small and/or single sex, non‐representative samples 19; or have a limited battery of questions assessing sexual health and satisfaction 13. The association between UI and sexual health amongst generally healthy, community‐dwelling individuals is less clear. A recent case‐control analysis using data from the multinational European Prospective Investigation into Cancer and Nutrition (EPIC) study found that subjects with OAB and other LUTS reported significantly lower sexual satisfaction, higher rates of depressive symptoms and erectile dysfunction compared with demographically matched controls 13.

We used data from Wave 6 of the English Longitudinal Study of Ageing (ELSA), a population‐representative panel survey of ageing, retirement and health in middle‐aged and older men and women living in England, to examine the association between UI and sexual health. We hypothesised that individuals reporting ‘any UI’ would have poorer sexual health in terms of a lower frequency of sexual activities and a higher prevalence of sexual difficulties and concerns, compared to those without UI, and there would be a dose severity between more sporadic vs persistent UI. We also hypothesised that if such associations were seen they would be attenuated, or perhaps fully explained, by sociodemographic differences, poorer physical and psychological health, and/or lifestyle factors.

## Subjects and Methods

### Participants and Study Design

The core data were from Wave 6 (2012/2013) of ELSA, a nationally representative longitudinal panel survey of community‐dwelling men and women aged ≥50 years in England 20. Data collection consisted of a face‐to‐face interview and self‐completion questionnaires. The general methods of data collection are detailed at: http://www.elsa-project.ac.uk. A total of 7079 (67%) participants completed and returned the paper‐based Sexual Relationships and Activities Questionnaire (SRA‐Q). We restricted this analysis to ELSA members who reported ‘any sexual activity’ in the last 12 months, had complete UI data at Wave 6, and for whom sampling probability weights were available (see below), leaving 3805 individuals in the main analysis sample. Item nonresponse was <1% for the sexual health variables included here. ELSA Wave 6 received ethical approval from the National Research Ethics Service Committee South Central ‐ Berkshire, UK, and all participants provided written informed consent.

### UI

Questions on current UI were asked as part of the face‐to‐face interview during Wave 6. Participants were asked two questions: (1) During the last 12 months, have you lost any amount of urine beyond your control? (yes/no); (2) When you had this problem, did it last for more than 1 month? (yes/no). Those who answered ‘yes’ to the first question but ‘no’ to the second were categorised as having ‘sporadic UI’; those answering ‘yes’ to both questions were categorised as having ‘persistent UI’. Participants provided this information at previous biennial waves of data collection (Waves 2–5), so using the first question only (During the last 12 months, have you lost any amount of urine beyond your control?) we generated an additional retrospective variable categorising those with ‘no reported history of UI’, ‘UI for ≤4 years’ or ‘UI for >4 years’.

### Sexual Activities, Problems and Concerns

The ELSA SRA‐Q included questions on frequency of sexual activities, sexual functioning and difficulties, concerns about sexual health, and changes in sexual health over the last 12 months. Table 1 summarises the items from the SRA‐Q used as dependent variables in this analysis. One question in the SRA‐Q asked about lifetime sexual experiences (5‐point scale: entirely opposite sex ‐to‐ entirely same sex) and this was recoded into two categories; ‘entirely opposite sex’ vs ‘some ‐to‐ entirely same sex’. Participants completed the SRA‐Q in private and returned it in a sealed envelope. The full range of sexuality measures assessed in the SRA‐Q and cross‐sectional associations with demographic, lifestyle, and health factors have been described previously 21. The SRA‐Q instrument is freely available at: http://www.elsa-project.ac.uk/documentation.

**Table 1:** Key sexual health variables used in the ELSA

Topic	Response set
Sexual behaviours/activities (…during the past month)	7‐point scale: ‘not at all’ to ‘more than once a day’. Women and men answering *2–3 times a month or more* were classified as participating frequently in that sexual behaviour/activity
How often did you think about sex?	
How many times have you had or attempted sexual intercourse (vaginal, anal or oral)?	
How frequently did you engage in other sexual activities (kissing, petting or fondling)?	
How often did you masturbate?	
Sexual functioning (…during the past month)	5‐point scale: ‘never’ to ‘always’. Women answering *never* or *much less than half the time* were classified as having difficulty becoming aroused.
How often did you feel sexually aroused during sexual activity?	
Are you able to get or keep an erection which would be good enough for sexual activity?	4‐point scale: ‘always able’ to ‘never able’. Men answering *never able* or *sometimes able* were classified as having erectile difficulties
When you had sexual stimulation how difficult was it for you to reach orgasm?	5‐point scale: ‘impossible’ to ‘not at all’. Women and men answering *moderately difficult* to *impossible* were classified as having orgasm difficulties
How often did you experience pain or vaginal dryness during sexual activity?	5‐point scale: ‘never’ to ‘always’. Women answering *usually* or *always* were classified as having pain or vaginal dryness difficulties
Change in sexual health (…compared with a year ago)	
Has your sexual drive/desire changed?	5‐point scale: ‘increased a lot’ to ‘decreased a lot’. Women and/or men answering *decreased moderately*, or *decreased a lot* were classified as having declines in that sexual behaviour/activity compared to a year ago
Has the overall frequency of your sexual activities changed?	
Has your ability to become sexually aroused changed?	
Has your ability to have an erection changed?	
Sexual concerns (…during the past month)	5‐point scale: ‘not at all worried or concerned’ to ‘extremely worried or concerned’. Women and/or men answering *moderately*,* very* or *extremely worried or concerned* were classified as being concerned about that particular sexual behaviour/activity
Have you been worried or concerned by your level of sexual desire?	
Have you been worried or concerned by the frequency of your sexual activities?	
Are you worried or concerned by your current ability to become sexually aroused?	
Have you been worried or concerned by your ability to have an erection?	

### Other Assessments

During the face‐to‐face interview participants were asked about their current living arrangements, the age at which they left education, and whether they had a doctor diagnosis of high blood pressure, heart conditions, arthritis, diabetes, asthma, chronic lung conditions, or if they had ever suffered a stroke or been diagnosed with cancer. A morbidity count variable was also created categorised as: 0, 1, 2, or ≥3 chronic conditions. Smoking status was recorded as ‘current’ or ‘non‐smoker’, and typical frequency of alcohol consumption over the past year as ‘never’ or ‘rarely’ (never–once or twice), ‘regularly’ (once every 2 months–twice a week) or ‘frequently’ (3 days a week–almost every day). Depressive symptoms were assessed using the eight‐item version of the Center for Epidemiologic Studies Depression scale (CES‐D), with a score of ≥4 indicative of likely depression 22.

### Statistical Analysis

Analyses were conducted using STATA SE v14.2 (StataCorp, College Station, TX). We used weights to correct for sampling probabilities and differential non‐response, including to the SRA‐Q, and to calibrate back to the 2011 Census population distributions for sex and age.

Specifically, these weights accounted for: (i) the differential probability of being included in the Wave 6 sample and (ii) for non‐response to the SRA‐Q instrument (full details available at: http://www.elsa-project.ac.uk). Logistic regressions adjusted for age, partner status, number of morbidities, age at which they left education, smoking status, and frequency of alcohol consumption were used to determine the association of both current UI status at Wave 6 (no UI, sporadic UI, persistent UI) and retrospective UI (no reported history of UI, UI for ≤4 years, UI for >4 years) with sexual activities, difficulties and concerns, separately by sex. A confounder analysis is presented in Appendix 1, supporting the rationale for the inclusion (or exclusion) of these additional adjustment variables in the final logistic regression models. Results were expressed as odds ratios (ORs) and 95% CIs.

## Results

Table 2 summarises the demographic, health, and lifestyle characteristics of the Wave 6 analysis sample who reported any sexual activity in the last 12 months. Women were less likely to have left education beyond 18 years of age and consumed alcohol less frequently, as compared to men (all *P* < 0.05). Women were more likely than men to report arthritis, whilst men were more likely to report high blood pressure, cardiovascular disease, and diabetes (all *P* < 0.05).

**Table 2:** Characteristics of ELSA Wave 6 analysis sample

Variable	Women (*n* = 1792)	Men (*n* = 2013)
Age, years, mean (SD)	63.4 (7.3)	64.7 (7.8)
Weighted % (95% CI)		
Partner status		
Married/cohabiting	77.2 (74.9, 79.5)	75.6 (73.2, 78.1)
Divorced/separated	13.3 (11.3, 15.2)	12.0 (10.0, 13.9)
Never married	4.6 (3.4, 5.8)	8.6 (6.9, 10.3)
Widowed	4.9 (3.9, 5.9)	3.8 (2.9, 4.7)
Lifetime sexual experience		
Entirely with opposite sex	94.9 (93.7, 96.2)	93.3 (91.8, 94.9)
Some‐to‐entirely with same sex	5.1 (3.8, 6.3)	6.7 (5.1, 8.2)
Age (years) left education		
≤14	3.9 (2.8, 5.0)	4.5 (3.6, 5.5)
15–18	74.9 (72.5, 77.3)	68.5 (66.0, 71.1)
≥19	21.2 (18.9, 23.5)	26.9 (24.4, 29.4)
Top five self‐reported chronic conditions		
High blood pressure*	29.9 (27.4, 32.4)	37.0 (34.5, 39.6)
Arthritis	35.9 (33.3, 38.5)	24.5 (22.4, 26.6)
Cardiovascular disease†	12.4 (10.6, 14.1)	17.1 (15.2, 19.0)
Diabetes*	6.5 (5.1, 7.9)	9.5 (8.1, 10.9)
Asthma*	9.4 (7.9, 11.0)	8.6 (7.2, 10.0)
Morbidity count		
0	38.2 (35.4, 40.9)	36.8 (34.1, 39.5)
1	33.9 (31.3, 36.5)	34.4 (31.9, 37.0)
2	17.9 (15.8, 19.9)	17.6 (15.8, 19.5)
≥3	10.1 (8.5, 11.6)	11.1 (9.6, 12.6)
Depression‡	13.4 (11.3, 15.5)	9.7 (7.9, 11.5)
Smoking status		
Current	13.0 (10.9, 15.0)	14.8 (12.7, 17.0)
Frequency of alcohol consumption§		
Never/rarely	29.6 (27.0, 32.1)	17.5 (15.3, 19.7)
Regularly	51.8 (49.0, 54.6)	58.0 (55.3, 60.6)
Very frequently	18.6 (16.6, 20.7)	24.5 (22.3, 26.7)

*Also includes self‐reported use of medications to manage these conditions. ^†^Heart conditions and/or stroke. ^‡^CES‐D (8‐item) score of ≥4. ^§^Frequency of alcohol consumption over the past year (never/rarely = never–once or twice, regularly = once every 2 months–twice a week, very frequently = 3 days a week–daily).

**Figure 1:**
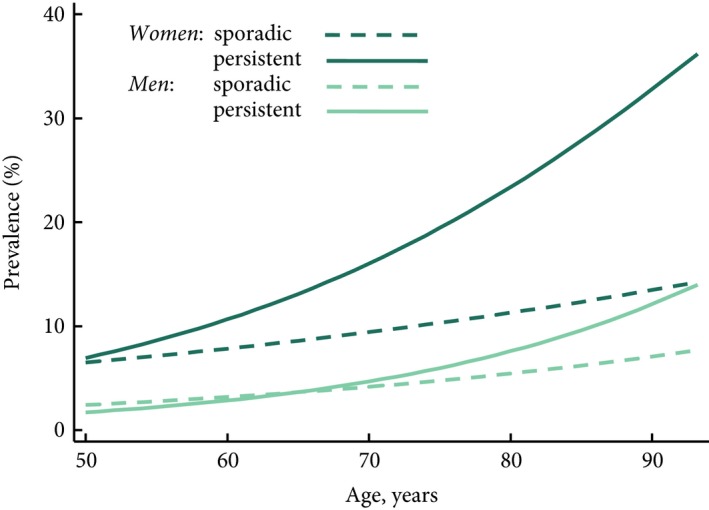
Weighted prevalence of reported UI over the past 12 months amongst ELSA Wave 6 analysis sample: by age and gender.

UI status at both Wave 6 and retrospectively (Waves 2–6) is shown in Table 3. Overall, 391 (20.0%) women and 141 (6.9%) men reported any UI in the last 12 months. Of those women reporting UI at Wave 6, 169 (41.4%) reported sporadic UI and 229 (58.6%) persistent UI; for men the proportions were 67 (47.5%) and 74 (52.5%), respectively. The prevalence of any reported UI at Wave 6 increased cross‐sectionally with age for both sexes (Fig. 1). A total of 3339 participants had complete retrospective UI data from Wave 2 to Wave 6, and of those women with any UI, 180 (50.4%) reported UI for ≤4 years and 177 (49.6%) UI for >4 years; for men the proportions were 92 (71.3%) and 37 (28.7%), respectively. The mean (SD) ages of the no UI, sporadic UI and persistent UI groups were: 62.9 (7.2), 64.1 (6.6) and 65.8 (8.1) years for women; 64.5 (7.7), 66.6 (9.6) and 67.2 (8.2) years for men, respectively. The only statistically significant age difference between groups was for the no UI vs the persistent UI group for both sexes (*P* < 0.05).

**Table 3:** UI status of ELSA analysis sample

Variable	Women	Men
UI in last 12 months (Wave 6)	(*n* = 1792)	(*n* = 2013)
Weighted % (95% CI)		
No UI	80.0 (77.9, 82.1)	93.1 (91.8, 94.5)
Sporadic UI	8.1 (6.7, 9.6)	3.4 (2.4, 4.5)
Persistent UI	11.9 (10.2, 13.6)	3.5 (2.6, 4.3)
UI in last 8 years (Waves 2–6)	(*n* = 1552)	(*n* = 1787)
Weighted % (95% CI)		
No reported UI	77.7 (74.8, 79.0)	92.8 (91.5, 93.9)
UI reported for ≤4 years	11.6 (10.1, 13.3)	5.1 (4.2, 6.3)
UI reported for >4 years	11.4 (9.9, 13.1)	2.1 (1.5, 2.8)

The sample size was smaller in the retrospective UI sample compared to the present UI sample due to case matching ELSA participants between Waves 2 and 6 (see http://www.elsa-project.ac.uk/timetable for details).

### UI and Any Sexual Activity

As reported previously, of the 6201 core ELSA members (excluding partners who were aged <50 years) with SRA‐Q data, 53.7% of women and 77.7% of men reported ‘any’ sexual activity in the last year 21. Although this analysis was restricted to those individuals who reported any sexual activity in the last 12 months, differences were seen between men and women with respect to any sexual activity and UI at Wave 6. Amongst women there was no significant association between either sporadic UI (OR 1.29, 95% CI 0.88, 1.91) or persistent UI (OR 1.27, 95% CI 0.96, 1.67) and the likelihood of reporting any sexual activity in the last 12 months. This relationship was different for men where, although no significant association was seen with sporadic UI (OR 1.51, 95% CI 0.78, 2.93), those reporting persistent UI (OR 0.49, 95% CI 0.28, 0.87) were less likely to report any sexual activity in the last 12 months compared to men without UI.

### Current UI and Sexual Health (ELSA Wave 6)

Associations between current UI and sexual activities, difficulties and concerns differed by both sex and whether UI was categorised as sporadic or persistent (Table 4). After adjustment for demographic, health, and lifestyle factors, compared to women with no UI, women with sporadic UI reported a 50% reduced odds of frequent sexual intercourse and a 74% increased odds of vaginal dryness. This group also reported significant declines in their frequency of sexual activities and their ability to become sexually aroused compared to a year ago, and was more concerned about the frequency of their sexual activities compared to women who were free of UI. Compared to women without UI, women with persistent UI reported a 56% increased odds of difficulty achieving orgasm and a 69% increased odds of declines in frequency of sexual activities over the last year. The persistent UI group were also significantly more concerned about both the frequency of their sexual activities and their ability to become sexually aroused.

**Table 4:** Association of current UI status with sexual health in women and men reporting ‘any’ sexual activity in the last 12 months. Weighted percentages and ORs (95% CIs). Referent for logistic regression models = No UI

Sexual health variables	*N*	No UI	Sporadic UI		Persistent UI	
		%	%	Adjusted OR (95% CI)	%	Adjusted OR (95% CI)
Women						
Frequently thinking about sex	1776	72.9	76.3	1.29 (0.84, 1.97)	71.5	1.32 (0.93, 1.88)
Frequent sexual intercourse	1770	52.8	34.8	0.50 (0.33, 0.76)**	44.7	0.90 (0.64, 1.27)
Frequent kissing, fondling and petting	1770	69.4	59.7	0.71 (0.47, 1.07)	64.7	0.94 (0.67, 1.33)
Frequent masturbation	1763	15.3	20.2	1.45 (0.86, 2.45)	18.1	1.57 (0.96, 2.55)
Difficulty becoming sexually aroused	1406	32.3	28.1	0.75 (0.46, 1.21)	35.1	0.94 (0.64, 1.37)
Difficulty achieving orgasm	1339	25.6	31.2	1.34 (0.82, 2.17)	35.6	1.56 (1.03, 2.37)*
Experience pain during sexual activity	1386	9.5	12.8	1.32 (0.70, 2.49)	13.3	1.33 (0.82, 2.17)
Experience vaginal dryness during sexual activity	1385	17.7	27.4	1.74 (1.09, 2.76)*	25.4	1.46 (0.97, 2.20)
Decline in sexual drive/desire over last year	1786	31.2	33.1	1.12 (0.74, 1.69)	39.8	1.33 (0.93, 1.92)
Decline in frequency of sexual activities over last year	1780	36.3	47.3	1.55 (1.04, 2.32)*	51.1	1.69 (1.20, 2.40)**
Decline in ability to become sexually aroused over last year	1397	24.6	37.4	1.83 (1.16, 2.89)**	32.5	1.32 (0.88, 1.98)
Concerned about current level of sexual desire	1787	10.8	7.1	0.71 (0.37, 1.38)	11.6	1.20 (0.73, 1.98)
Concerned about current frequency of sexual activities	1779	7.2	7.7	1.55 (1.04, 2.32)*	10.3	1.69 (1.20, 2.40)**
Concerned about current ability to become sexually aroused	1398	6.3	8.7	1.56 (0.75, 3.21)	15.1	2.47 (1.38, 4.41)**
Men						
Frequently thinking about sex	2004	93.0	90.8	1.11 (0.24, 5.17)	92.5	1.41 (0.44, 4.57)
Frequent sexual intercourse	1994	50.1	40.3	0.86 (0.45, 1.65)	30.9	0.58 (0.32, 1.04)
Frequent kissing, fondling and petting	1995	64.5	54.1	0.88 (0.48, 1.61)	59.1	0.97 (0.55, 1.70)
Frequent masturbation	1994	45.1	42.0	0.90 (0.37, 2.19)	43.1	1.33 (0.78, 2.27)
Erectile difficulties	2000	26.4	34.1	0.95 (0.52, 1.73)	61.4	2.73 (1.44, 5.18)**
Difficulty achieving orgasm	1734	13.7	15.1	0.69 (0.26, 1.84)	35.4	2.01 (1.07, 3.81)*
Decline in sexual drive/desire over last year	2008	27.6	48.2	2.26 (1.19, 4.29)*	46.0	1.75 (1.01, 3.06)*
Decline in frequency of sexual activities over last year	2004	35.9	55.0	2.01 (1.05, 3.87)*	46.7	1.36 (0.78, 2.38)
Decline in ability to have an erection over last year	2007	22.8	39.1	1.81 (0.83, 3.94)	45.9	2.06 (1.17, 3.61)*
Concerned about current level of sexual desire	2009	13.9	33.3	2.70 (1.29, 5.65)**	32.9	2.20 (1.24, 3.90)**
Concerned about current frequency of sexual activities	2002	12.2	18.9	1.55 (1.04, 2.32)*	31.3	1.69 (1.20, 2.40)**
Concerned about current ability to have an erection	2009	12.5	25.3	1.83 (0.81, 4.15)	37.3	2.56 (1.33, 4.92)**

Weighted logistic regressions adjusted for age, partner status, age left education, morbidity count, depression, smoking status and frequency of alcohol consumption. The denominator varies due to questionnaire routing (see http://www.elsa-project.ac.uk for details) and some participants declining to answer some questions. *P < 0.05; **P < 0.01.

Compared to men free from UI, men with sporadic UI reported 126% and 101% increased odds of declines in both their sexual desire and frequency of sexual activities over the last year (Table 4). Men with sporadic UI were also more concerned about their sexual desire and their frequency of sexual activities. More significant associations were seen in men with persistent UI where, compared to men without UI, they additionally reported increased erectile and orgasm difficulties, declines in their ability to have an erection, and concerns about their current ability to have an erection.

### Retrospective UI and Sexual Health (ELSA Waves 2–6)

Associations between sexual health and UI were related to UI duration in both sexes. Women who reported UI for ≤4 years had a 40% reduction in odds of frequent sexual intercourse, a 102% increase in odds of frequent masturbation, and a 92% increase in odds of experiencing vaginal dryness compared with women free of UI (Table 5). Women with UI for ≤4 years also reported significant declines in their frequency of sexual activity and their ability to become sexually aroused over the last year. The only significant association amongst women with UI for >4 years was an increased odds of being concerned about their current ability to become sexually aroused.

**Table 5:** Association of retrospective UI status with sexual health in women and men reporting ‘any’ sexual activity in the last 12 months. Weighted percentages and ORs (95% CIs). Referent for logistic regression models = No UI

Sexual health variables	*N*	No UI	UI (≤4 years)		UI (>4 years)	
		%	%	Adjusted OR (95% CI)	%	Adjusted OR (95% CI)
Women						
Frequently thinking about sex	1538	68.6	66.9	1.05 (0.72, 2.15)	71.8	1.46 (0.99, 2.15)
Frequent sexual intercourse	1531	49.2	35.9	0.60 (0.41, 0.86)**	37.5	0.78 (0.54, 1.12)
Frequent kissing, fondling and petting	1532	66.1	60.6	0.83 (0.58, 1.20)	60.8	0.94 (0.65, 1.37)
Frequent masturbation	1528	11.7	20.1	2.02 (1.28, 3.20)**	13.3	1.16 (0.72, 1.88)
Difficulty becoming sexually aroused	1197	37.6	37.5	0.94 (0.63, 1.39)	36.5	0.81 (0.53, 1.24)
Difficulty achieving orgasm	1134	29.6	37.2	1.43 (0.92, 2.23)	35.8	1.33 (0.85, 2.09)
Experience pain during sexual activity	1179	10.4	16.9	1.66 (0.98, 2.83)	16.1	1.55 (0.84, 2.86)
Experience vaginal dryness during sexual activity	1178	20.8	33.8	1.92 (1.26, 2.93)**	29.1	1.50 (0.96, 2.37)
Decline in sexual drive/desire over last year	1547	30.5	36.3	1.30 (0.90, 1.87)	37.9	1.33 (0.90, 1.97)
Decline in frequency of sexual activities over last year	1541	37.3	46.8	1.46 (1.02, 2.07)*	44.6	1.23 (0.84, 1.80)
Decline in ability to become sexually aroused over last year	1397	26.4	32.1	1.79 (1.13, 2.82)*	37.0	1.43 (0.97, 2.13)
Concerned about current level of sexual desire	1547	9.0	8.8	1.02 (0.58, 1.79)	9.2	1.06 (0.60, 1.90)
Concerned about current frequency of sexual activities	1540	6.1	6.7	1.14 (0.59, 2.20)	5.5	0.86 (0.42, 1.79)
Concerned about current ability to become sexually aroused	1190	6.4	10.5	1.74 (0.91, 3.34)	15.1	2.46 (1.29, 4.67)**
Men						
Frequently thinking about sex	1742	90.6	88.9	1.06 (0.43, 2.58)	–†	–†
Frequent sexual intercourse	1769	43.8	32.7	0.74 (0.45, 1.22)	17.5	0.35 (0.14, 0.89)*
Frequent kissing, fondling and petting	1770	61.7	61.4	1.26 (0.77, 2.05)	38.3	0.50 (0.24, 1.02)
Frequent masturbation	1770	40.0	38.7	1.08 (0.65, 1.77)	38.5	1.14 (0.55, 2.39)
Erectile difficulties	1775	32.9	50.6	1.60 (0.93, 2.75)	67.4	2.59 (1.06, 6.33)*
Difficulty achieving orgasm	1522	16.9	27.5	1.38 (0.72, 2.65)	35.2	1.33 (0.53, 3.36)
Decline in sexual drive/desire over last year	1782	30.0	45.1	1.70 (1.04, 2.77)*	52.6	1.93 (0.92, 4.07)
Decline in frequency of sexual activities over last year	1779	37.1	52.9	1.79 (1.10, 2.90)*	41.1	1.09 (0.51, 2.33)
Decline in ability to have an erection over last year	1782	26.5	43.5	1.86 (1.14, 3.04)*	37.5	1.17 (0.49, 2.82)
Concerned about current level of sexual desire	1783	13.9	27.0	2.00 (1.18, 3.39)*	32.1	2.41 (1.08, 5.36)**
Concerned about current frequency of sexual activities	1777	12.7	22.3	1.79 (1.01, 3.16)*	16.2	1.31 (0.52, 3.32)
Concerned about current ability to have an erection	1783	15.1	24.6	1.48 (0.86, 2.54)	38.7	2.50 (1.17, 5.35)*

Weighted logistic regressions adjusted for age, partner status, age left education, morbidity count, depression, smoking status and frequency of alcohol consumption. The denominator varies due to questionnaire routing (see http://www.elsa-project.ac.uk for details) and some participants declining to answer some questions. ^†^No observations in this group. *P < 0.05; **P < 0.01.

Compared to men with no UI, those with UI for ≤4 years had 70%, 79% and 80% increased odds of declines in their sexual desire, frequency of sexual activity, and their ability to have an erection over the last year, respectively (Table 5). Men in this group also reported increased concerns about both their current level of sexual desire and current frequency of sexual activities. Significant associations amongst men with UI for >4 years included a 65% reduction in odds of frequent sexual intercourse and a 159% increased odds of reporting erectile difficulties. Men with UI for >4 years also reported increased concerns about both their current level of sexual desire and current ability to have an erection.

## Discussion

These nationally representative data reveal associations between UI and poorer sexual health amongst older women and men in England. Reported UI was most consistently associated with greater declines in sexual activity and function compared to a year ago, and concerns about frequency of sexual activities and sexual functioning. Cross‐sectionally, significant associations between persistent UI and current difficulties achieving orgasm were seen in both sexes, although functional difficulties in women (vaginal dryness) and in men (erectile difficulties) showed differential relationships with sporadic and persistent UI, respectively. Retrospective reports of UI showed broadly similar associations with sexual health as seen cross‐sectionally, although women with UI for ≤4 years reported more frequent masturbation and men with UI >4 years reported less frequent sexual intercourse.

Our present findings build on previous work showing that UI and other LUTS can have a marked negative effect on sexual health and satisfaction. Using data from the population‐based EPIC study, Coyne et al. 13 found that OAB and other LUTS had multidimensional impacts on older men and women (mean age 54 years), including worse health‐related QoL, higher rates of depression, and decreased enjoyment of sexual activity. The authors also found that higher rates of erectile dysfunction and decreased sexual satisfaction were most strongly associated with OAB plus voiding symptoms as compared to OAB alone or OAB with UI alone 13. Although the EPIC data included detailed self‐report items covering different aspects of UI, the questions about sexual health were more limited than those captured in ELSA. In a Danish survey of >7000 participants aged 40–65 years, Hansen 23 reported that LUTS was independently associated with erection problems and satisfaction with sex life in men and sexual function in women. Similarly, Salonia et al. 19 found that amongst a sample of Italian women (age range 19–66 years), those reporting UI or LUTS also complained of sexual dysfunctions in a significantly higher number than a general, healthy female population without urinary symptoms. Our present data extend the upper age limits around one to two decades above that typically included in other population‐based studies, whilst also capturing detailed information about sexual activities, problems, and satisfaction.

Our present cross‐sectional findings did not show a consistent pattern of association between sporadic or persistent UI and the frequency of sexual activities and sexual problems in both women and men. More consistent associations were seen for declines in sexual activities and function over the last year, and concerns about sexual activities and function. It was interesting that UI was consistently associated with concerns about sexual desire, frequency of sexual activities, and sexual function, particularly amongst men. We do not know the relative severity of self‐reported UI in ELSA and it is possible that some participants may not perceive their UI as severe enough to seek treatment. In addition, we do not have any longitudinal data on sexual health to clarify whether those reporting UI at Wave 6 were more or less sexually active in the past than those reporting no UI. Nonetheless, individuals with UI were more likely to report greater sexual concerns and retrospective reports of declines in sexual activity and functioning, if not so marked impacts on current sexual activities.

The physiological, psychological and/or social mechanisms linking UI and sexual health remain poorly defined and we were unable to directly investigate them here. It seems likely that common, coexistent risk factors are, at least in part, mediating the observed associations between UI and sexual health. For example, we found that depression was strongly associated with both UI and poorer sexual health (Appendix 1), and it is plausible that this in turn may be partly driven by detrimental effects of UI on QoL due to leakage, odour, anxiety, and embarrassment. Although we were unable to define whether the observed associations between UI and sexual outcomes were primarily due to common risk factors, or if the experience of UI results in sexual problems, these factors are likely to have important ramifications in a clinical setting with regard to preferred treatment options and optimal outcomes. For more explicitly gynaecological/urological factors, ELSA has gathered self‐report information on hysterectomy, oophorectomy, and diagnosed prostate cancer (but no measure of BPH). However, additionally adjusting the logistic regression models for these obstetric events or prostate disease (or dropping affected cases) did not substantively change the reported results (data not shown). We also found that including self‐reported lifetime sexual experiences (‘entirely opposite sex’ vs ‘some ‐to‐ entirely same sex’) in the logistic regression models had no substantive effect on either the magnitude or significance of the observed associations (data not shown).

A somewhat surprising finding with the present retrospective data was that participants reporting UI for ≤4 years had a higher number of significant associations with poorer sexual health than those with UI for >4 years. This was particularly evident amongst women, and perhaps reflects a ‘response shift’ model of adaptation to illness, i.e., chronic UI, whereby internal standards, values, and subjective perceptions of QoL change over time 24. Those individuals who may have lived with UI for shorter periods of time (UI for ≤4 years) may have had less opportunity to adapt it into their daily lives as compared to those who had lived with UI for longer (UI for >4 years) and, as a result, reported more sexual problems. This idea of a response‐shift paradigm was also supported, at least in women, by the observation that declines over the last year in the frequency of sexual activities and the ability to become sexually aroused were only seen amongst women reporting UI for ≤4 years. Additionally, whilst the cross‐sectional analyses support our first hypothesis that UI would be associated with poorer sexual health in both women and men, we did not consistently find that those individuals with persistent UI had uniformly worse sexual health than those who reported sporadic UI. It is plausible that sporadic UI may be more reflective of new onset of UI problems, although we were unable to define other pre‐existing characteristics, including personality factors that may have influenced any response shift reflected in our analyses. A further surprising observation was that women with UI for ≤4 years reported more frequent masturbation than those with no UI or UI for >4 years. Although we can only speculate that this also reflects a response‐shift adaptation, we can hypothesise that this reveals a short‐term shift to more solo sexual activities. Thus, ongoing sexual desires could be met, whilst avoiding potential barriers to partnered sexual activity related to embarrassment and anxiety. It is also possible that the ‘UI for ≤4 years’ vs ‘UI for >4 years’ grouping is related to age of first UI incidence. However, given the wide age range of the ELSA cohort we are unable to definitively assess time of first UI incidence to examine if earlier or later onset UI is differentially associated with specific aspects of sexual health, and whether age cohort factors (multiple chronic conditions, partner availability) also influence these associations.

The ELSA study has unique positive features; the data were derived from a large and representative community‐dwelling sample covering midlife to the oldest old, who were not recruited explicitly to answer questions on their sexual health. The ELSA dataset, however, presented a contrary limitation compared to the EPIC study 13; whilst we were able to investigate a wide variety of sexual health outcomes we were unable to draw more nuanced distinctions between underlying UI aetiologies. The ELSA questions referred solely to UI and did not attempt to differentiate between UI subtypes (OAB, stress UI, other LUTS etc.). Further exploration of potential differential relationships across different subtypes of UI would require more exhaustive questionnaire items than the ELSA schedule allows. However, future collection of population‐representative data including UI typology, frequency and volume of urine loss, and duration of UI would allow for a more thorough examination of associations, not only with sexual function and satisfaction, but also with QoL and ageing‐related health issues. We were also unable to meaningfully assess the degree to which self‐reported UI was reversible or persistent. This a potentially important limitation, given that reversible UI may be caused by distinct (but short‐term) factors, such as UTIs or faecal impaction, and in turn may only impact transiently on sexual function. ELSA did not oversample ethnic (2.1% of the analysis sample identified as non‐White) or sexual minority groups and the results presented here may not be generalisable to ethnic minority, and lesbian, gay and other groups who do not identify themselves as heterosexual. Our present data were self‐reported and, although the interview methods have accepted validity 25 we cannot exclude reporting bias. Those ELSA participants who chose not to complete the SRA‐Q may not have done so due to pre‐existing sexual problems and/or feeling that they were ‘retired’ from sex. The non‐response weights will have partly dealt with this, with weighting for non‐response to the SRA‐Q directly accounting for the variation in response according to demographic and health characteristics. However, these weights would not have dealt with UI and/or sexual health characteristics that were unrelated to the factors used for weighting, resulting in our sample being potentially biased towards more functionally and cognitively fit older people. Our present data, therefore, may have overestimated the prevalence of sexual activities, particularly amongst the oldest‐old, and potentially underestimated the prevalence of UI and sexual problems. This latter point is of particular importance when we consider that both UI and sexual health are taboo subjects in society, and likely to be under‐reported 26, 27. Finally, the cross‐sectional data preclude any examination of the temporal nature of the observed associations between UI and sexual health.

In conclusion, UI was negatively associated with aspects of sexual health in both women and men, mainly influencing self‐reported declines in sexual activity and function, and elevated levels of sexual concerns. Given the relatively high prevalence of UI, particularly amongst women, clinicians should be aware of the potential impacts on QoL and recognise that sexual activity and satisfaction are key factors in this equation. Both UI and sexual problems are likely to be under‐reported by older people, reinforcing the need to ask about sexual function in clinical assessment, given the ‘taboo’ nature of both subjects. An increased recognition of the comorbid relationship between UI and sexual health could result in more inclusive treatment options being offered to improve both outcomes, potentially leading to more satisfying intimate lives.

## Conflict of Interest

None declared.

**Abbreviations**
CES‐D: Center for Epidemiologic Studies Depression scale
ELSA: English Longitudinal Study of Ageing
EPIC: European Prospective Investigation into Cancer and Nutrition
OAB: overactive bladder
OR: odds ratios
QoL: quality of life
SRA‐Q: Sexual Relationships and Activities Questionnaire
UI: urinary incontinence

## Appendix 1 The association between urinary incontinence (UI)/any sexual activity in the last 12 months and the adjustment variables included in the logistic regression analyses (Table 4).

**Table:**

Adjustment variables	Any reported UI in the last 12 months	Any sexual activity in the last 12 months
	OR (95% CI)	OR (95% CI)
Age (years)	1.02 (1.01, 1.03)***	0.90 (0.89, 0.91)***
Partner status		
Married/cohabiting	Reference	Reference
Divorced/separated	1.50 (1.24, 1.81)***	0.45 (0.39, 0.52)***
Never married	0.88 (0.65, 1.19)	0.56 (0.45, 0.70)***
Widowed	1.67 (1.40, 2.00)***	0.13 (0.11, 0.15)***
Lifetime sexual experience (women)		
Entirely with men	Reference	Reference
Some‐to‐entirely with women	1.50 (0.94, 2.41)	1.04 (0.77, 1.40)
Lifetime sexual experience (men)		
Entirely with women	Reference	Reference
Some‐to‐entirely with men	1.55 (0.83, 2.89)	1.40 (0.94, 2.11)
Age (years) left education		
≤14	Reference	Reference
15–18	0.70 (0.56, 0.88)**	3.18 (2.62, 3.86)***
≥19	0.62 (0.48, 0.80)***	5.91 (4.74, 7.38)***
Morbidity count		
0	Reference	Reference
1	1.22 (1.02, 1.47)*	0.71 (0.62, 0.81)***
2	1.67 (1.39, 2.02)***	0.41 (0.36, 0.48)***
≥3	2.44 (2.02, 2.96)***	0.28 (0.24, 0.33)***
Depression^†^		
No	Reference	Reference
Yes	2.20 (1.85, 2.60)***	0.52 (0.45, 0.60)***
Smoking status		
Never/ex‐smoker	Reference	Reference
Current smoker	1.03 (0.85, 1.25)	0.79 (0.68, 0.92)**
Frequency of alcohol consumption‡		
Never/rarely	Reference	Reference
Regularly	0.60 (0.52, 0.70)***	2.74 (2.44, 3.08)***
Very frequently	0.55 (0.46, 0.66)***	3.08 (2.65, 3.57)***

Note

*NB*: The above logistic regression included both participants reporting ‘any’ sexual activity in the past year and those reporting ‘no’ sexual activity in the past year. The denominator varied from *n* = 6727 to *n* = 6934 depending on missing data for the various covariates. ^†^CES‐D (8‐item) score of ≥4; ^‡^Frequency of alcohol consumption over the past year (never/rarely = never–once or twice, regularly = once every 2 months–twice a week, very frequently = 3 days a week–daily). **P* ≤ 0.05, ***P* ≤ 0.01, ****P* ≤ 0.001.
